# Surges in trematode prevalence linked to centennial-scale flooding events in the Adriatic

**DOI:** 10.1038/s41598-017-05979-6

**Published:** 2017-07-18

**Authors:** Daniele Scarponi, Michele Azzarone, Michał Kowalewski, John Warren Huntley

**Affiliations:** 1 Dipartimento di Scienze Biologiche, Geologiche e Ambientali, University of Bologna, via Selmi 3, Bologna, I-40126 Italy; 2 Florida Museum of Natural History, University of Florida, 1659 Museum Rd., Gainesville, FL 32611 USA; 3 Department of Geological Sciences, University of Missouri, 101 Geology Building, Columbia, MO 65211 USA

## Abstract

The forecasts of increasing global temperature and sea level rise have led to concern about the response of parasites to anthropogenic climate change. Whereas ecological studies of parasite response to environmental shifts are necessarily limited to short time scales, the fossil record can potentially provide a quantitative archive of long-term ecological responses to past climate transitions. Here, we document multi-centennial scale changes in prevalence of trematodes infesting the bivalve host *Abra segmentum* through multiple sea-level fluctuations preserved in brackish Holocene deposits of the Po Plain, Italy. Prevalence values were significantly elevated (p < 0.01) in samples associated with flooding surfaces, yet the temporal trends of parasite prevalence and host shell length, cannot be explained by Waltherian facies change, host availability, salinity, diversity, turnover, or community structure. The observed surges in parasite prevalence during past flooding events indicate that the ongoing global warming and sea-level rise will lead to significant intensification of trematode parasitism, suppressed fecundity of common benthic organisms, and negative impacts on marine ecosystems, ecosystem services, and, eventually, to human well-being.

## Introduction

Understanding the historical impact of climate variability on heterocious parasites is prerequisite for forecasting parasite-host interactions in the near future and assessing the potential implications for ecosystem health, ecosystem services, and human well-being^1–6^. However, ecological research on the response of parasites to anthropogenic warming is necessarily limited to short time scales of the most recent months and years^7, 8^. In this respect, the most promising avenue is offered by the latest Quaternary fossil record where, thanks to highly-resolved sequence and chrono-stratigraphic frameworks^9–16^, past parasite-host interactions can be examined over geologically short (10^2^–10^3^ years), societally relevant time scales.

**Figure 1:**
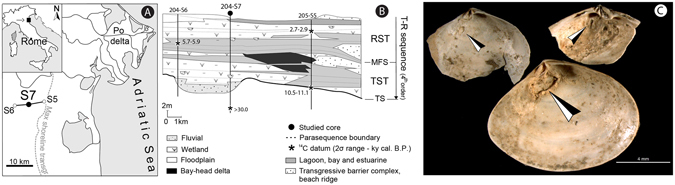
Location map, cross-section, and images of parasitized *Abra segmentum* valves. (**A**) Location map of investigated Po coastal plain sector, Italy (**B**) Schematic cross section (along dip) illustrating the stratigraphic stacking of facies across the investigated portion of the Holocene post-Last Glacial Maximum T-R sequence and location of the 204-S7 core. (**C**) Photomicrographs of *A. segmentum* with trematode-induced pits (black/white arrows). TS: transgressive surface, TST: transgressive systems tract, MFS: maximum flooding surface, RST: regressive systems tract, T-R: transgressive-regressive.

Digenean trematodes typically display a complex lifecycle with three hosts. The first intermediate host, where the parasite performs asexual reproduction of larvae (cercariae), is always a mollusk species. The newly emerged cercariae larvae infest the second intermediate host where the parasite is in an encysted, latent stage (metacercaria), waiting to be ingested by the third, definitive host, which is always a vertebrate organism that enables sexual reproduction of the adult parasite. A peculiarity of many trematode species, within the family Gymnophallidae, is that they have the same first and second intermediate individual hosts, skipping the intermediary, free-living cercariae stage^15^. It is usually in the second intermediate host stage that digeneans affect shell secretion in their molluscan hosts. Gymnophallids induce the active growth of characteristic pits with raised rims on the interior of their bivalve host’s shells (Fig. 1)^17–19^ and schistosomatids and echinostomatids may alter the geochemical composition of their host’s shells^20, 21^. Gymnophallid-induced pits are known from live-collected bivalve hosts and are readily preserved in the fossil record, providing a proxy for infestation by microscopic, non-biomineralized parasites^22^.

Previous quantitative analysis of a 9.6 ky record of Holocene estuarine deposits of the Pearl River^23^ demonstrated that trematode prevalence peaked in the lower part of paralic transgressive deposits recording the generalized inundation of the regional coastal system coincident with Meltwater Pulse 1c, that took place between 9.5 and 9.2 ky^24^. Similarly, significantly higher trematode prevalence was documented in host taxa from sediment-starved northern Adriatic strandline death assemblages, relative to that documented from comparable assemblages from the Po delta shoreline^25^. These two coastal regimes serve respectively as modern analogues for Holocene transgressive and prograding settings^26^. This putative link between overall sea-level rise and prevalence, if demonstrated on societally relevant time scales, could serve as an analogue for the response of parasitism to global warming in the coming decades to centuries. Here we explicitly test the link between short term (10^2^–10^3^ years) flooding pulses and upsurges in parasite prevalence using the fossil record of bivalve hosts from a cored Holocene back-barrier succession (Fig. 1; Po coastal plain, Italy). Additionally, we test for correlative relationships between parasite prevalence and other environmental and ecological factors to identify or rule out driving factors of this pattern.

### Coastal Po Plain Succession

**Figure 2:**
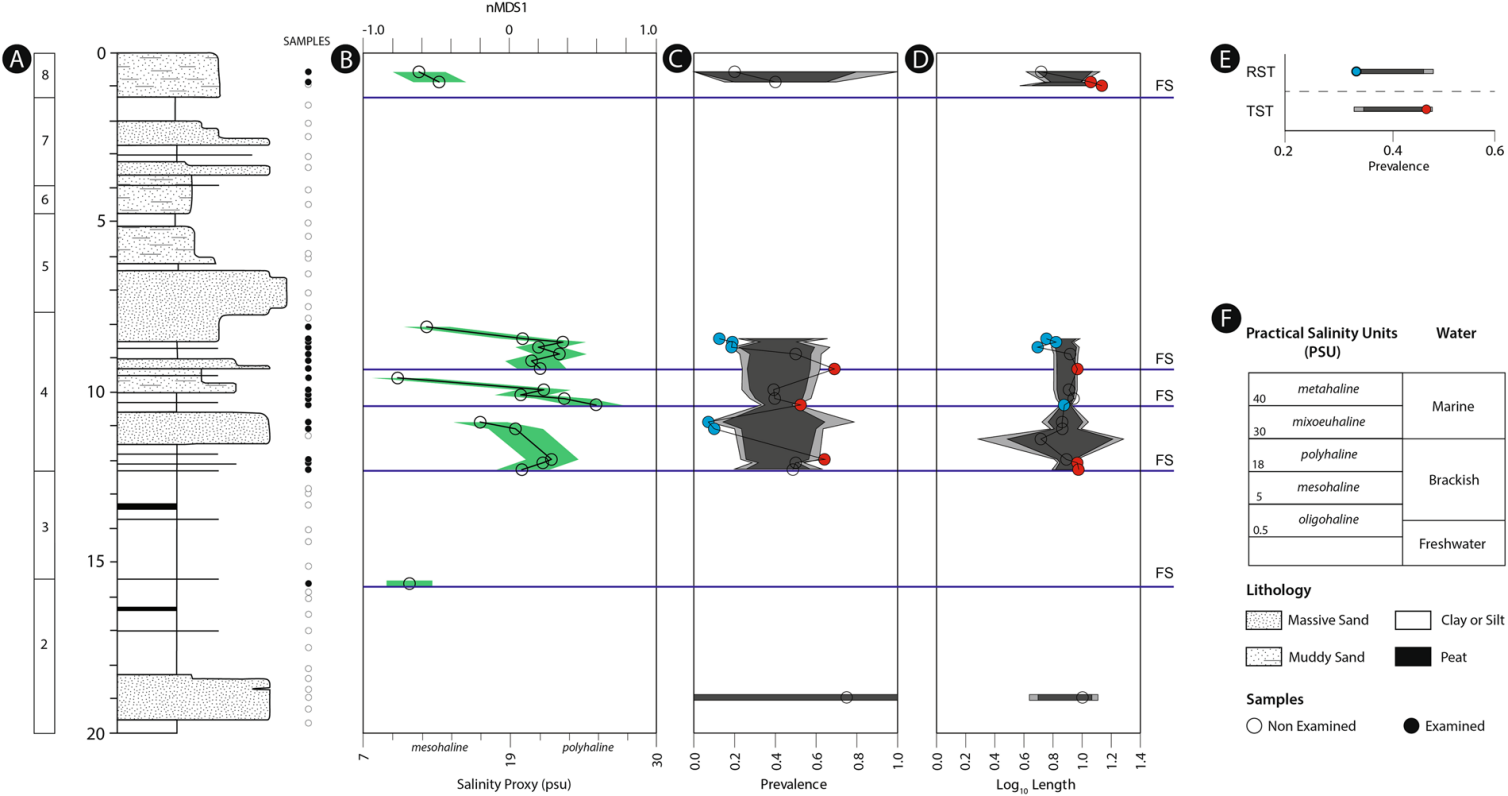
Detailed architectural, stratigraphic and bio-sedimentary (nMDS, *A. segmentum* trematode prevalence and shell length) features of core 204-S7. (**A**) Detailed stratigraphic column with 8 small-scale (millennial-scale sedimentary packages—parasequences defined in ref. 27 and the position of the studied samples: hollow circles represent samples with fewer than 15 specimens. (**B**) nMDS derived salinity trends along core highlighting back-barrier small-scale sedimentary packages and their internal architecture. Green field represents Standard Error of the Estimate (SEE) resulting from the RMA regression (see Extended Data Fig. S5) of nMDS derived salinity trends. (**C**) Prevalence of trematode pits among single samples recording more than 4 valves of *Abra segmentum*. (**D**) Mean log_10_-transformed anterior-posterior length of single samples of *A. segmentum*. (**E**) Prevalence values of data pooled by TST and RST (defined by nMDS derived trend along core). (**F**) Reference scale for Practical Salinity Units (PSU) with associated type of water and legend for lithology and samples. Dark and light grey fields on panels C, D, and E represent 95% and 99% confidence intervals (CI) derived from 10,000 iteration randomizations, respectively. Red circles indicate values greater than 95% CI, blue circles indicate values less than 95% CI, and hollow circles (in panels C and D) indicate values that fall within 95% CI. FS indicates back barrier correlative of flooding surfaces; nMDS: non Metric Multidimensional Scaling.

The post-Last Glacial Maximum (post-LGM) transgressive-regressive sequence of the coastal Po Plain is a wedge-shaped genetically related package of latest Pleistocene-to-Holocene strata. In the studied core 204-S7, the T-R sequence is a few tens of meters thick (Fig. 1), with its base defined by the transgressive surface resting on top of a weakly developed, Younger Dryas age paleosol (Online Methods). Several higher-order depositional cycles (parasequences), defined by their characteristic bounding surfaces, internal stacking patterns, and geometric relations to surrounding strata, characterize the internal structure of the post-LGM sequence (Fig. 1)^27^. These parasequences record high-frequency shifts in the local sea-level^28, 29^ and a high-resolution chronostratigraphic framework indicates that they formed on millennial (and shorter) time scales (Online Methods). The strata in core 204-S7, from bottom to top, are composed of stacked fluvial channel facies associations (>9 ky; parasequence 1 in ref. 27), passing upwards into poorly-drained floodplain/wetland facies alternations, overlain by brackish (lagoon/estuary) and thinning upward swamp facies associations (parasequences 2-4; Figs 1A,B and 2B, Extended Data Fig. 1). The subsequent middle-to-late Holocene (parasequences 5-7; Fig. 2) record a mosaic of floodplain and wetland deposits (lower delta plain; <6 ky), which are overlain by parasequence 8 that details renewed brackish settings related to the most recent shift of the Po delta toward its present position^27^.

## Results

We collected 61 bulk samples from the top 20 meters of core 204-S7 (Fig. 2A; Online Methods). Forty-five out of 61 samples contained mollusk fossils (Extended Data Fig. 1), resulting in a matrix of 3,151 individuals from 26 genera and 31 species (Online Methods; Extended Data Table S1). A non-metric Multidimensional Scaling (nMDS) ordination displays a pronounced gradient with species distributed along the nMDS1 axis according to their salinity tolerance (Online Methods; Extended Table S2). These results were robust to a variety of filters and other ordination types (see Online Methods; Extended Data Figs 3 and 4; Table S3). The resulting nMDS1 sample scores positively correlate with preferred salinity values for extant taxa in modern ecosystems (see Extended Data Fig. 3 for taxon salinity data; Tables S3 and S4), and are, therefore, a proxy for salinity (Online Methods), a common ecological driver in back barrier settings^30^.

The temporal trend of nMDS1 sample scores displays multiple orders of cyclicity. At the overall scale of the sedimentary package (Fig. 2B), the scores support the control of glacio-eustatic forcing on the development of the post-LGM sequence. Specifically, samples from parasequence one to four record the landward increasing influence of the Adriatic sea, while from parasequence four onward, the general trend toward lower salinity values is consistent with Po deltaic progradation into the Adriatic Sea (Figs 1B and 2A,B). At a higher resolution, the stratigraphic trajectory of nMDS1 sample scores highlights five flooding pulses depicted by major increases in salinity (in accordance with parasequence bounding surfaces of ref. 27), followed by a gradual return to reduced salinities (Fig. 2B). These abrupt and major salinity shifts are interpreted to represent non-Waltherian facies dislocations, with the overlying facies recording increased marine influence rather than a simple lateral shift to an adjacent environment relative to the underlying facies. The three salinity shifts recognized at 15.5, 12.3, and 1.1 m core depth (Fig. 2A) represent parasequence bounding surfaces developed over millennial time scales, whereas the remaining two are interpreted as higher frequency, centennial-scale pulses^27^ representing short-lived, rapid transition from mesohaline to polyhaline dominated environments (Fig. 2B). Accordingly, parasequence 4, which marks the turnaround from retrogradation to progradation (Fig. 2B), consists of a set of three higher frequency (centennial scale) units bounded by stratigraphically significant (i.e., non-Waltherian) shifts of facies (Fig. 2B).

The thin-valved *Abra segmentum*–a genus which is parasitized by *Parvatrema rebecqui* in modern environments^31^ – is the dominant species in core 204-S7 and displays a high prevalence of trematode-induced pits (34.4%; 348 infested of the total 1,012 valves). Anterior-posterior length of *A. segmentum* ranged between 1.5 and 22.3 mm (Extended Data Fig. 6). Single sample prevalence values range from 7.1% to 75.0% and display significant temporal variation (Fig. 2; Extended Data Table S4). At the systems tract level (i.e., a multi-millennial observational scale), there was a significant (i.e., outside the 95% confidence bounds estimated via randomization) difference in trematode prevalence values between the TST (44.9%) and RST (34.2%; Fig. 2E), consistent with previous findings^23, 26^. At the parasequence level, significantly elevated prevalence estimates are located in proximity to the millennial-scale flooding surface at 12.3 m and the centennial-scale flooding surfaces at 9.3 and 10.3 m (Fig. 2A,B). Significantly depressed prevalence estimates all occurred within these small-scale units (Fig. 2C).

## Discussion

The results demonstrate a repeated association between significantly elevated prevalence and centennial scale flooding events, support the link between sea-level rise and increasing trematode activity, and can serve as historical analogues for ongoing and future anthropogenic climate change. Despite evidence for the consistent relationship between transgression and trematode prevalence, it is doubtful that a relative rise in sea level alone drove this pattern. Many factors that can influence the biota, including temperature, nutrient availability, salinity, host availability, diversity, and community structure, co-vary with sea level changes and should be tested as driving factors^32, 33^. Increasing temperature has been shown to increase reproductive output and infectivity of a diverse array of pathogens and parasites^3, 4^ (but see refs 7, 8 and 34). As parasites derive nutrition from their hosts, it is not clear that changes in nutrient availability/productivity would directly control their distribution, however biological diversity is often related to productivity and its mode of delivery across a variety of scales and systems^35, 36^. Diversity and productivity often increase in concert until a tipping point above which diversity begins to decline, varying with the influence of consumers and disturbance level^37^. In this way productivity could control the distribution and abundance of many taxa that might serve as intermediate or definitive hosts, though likely in a non-linear manner. Salinity is a primary environmental driver of mollusk turnover in the studied system (Fig. 2B), and free-swimming larval (cercaria) production and survival time tend to decrease significantly in lowered salinity regimes in paralic environments; thereby reducing infestation of intermediate and/or definitive hosts^38, 39^. Here, however, as in ref. 23, the lack of correlation between salinity proxy and prevalence estimates (Table 1) suggests that salinity is not a strong driving factor of trematode prevalence at this spatial and temporal scale of observation.

**Table 1:** Spearman rank correlation coefficients and *p*-values (when *p* < α = 0.05; otherwise indicated as ns: non-significant) between arcsine-transformed trematode prevalence values of *Abra segmentum* from the 204-S7 core; nMDS1 sample scores; and environmental, ecological, and taphonomic variables. nMDS1: Non metric Multidimensional Scaling axis 1.

	Arcsine Prevalence	nMDS1
*A. segmentum* abundance	ns	R = +0.73, *p* = 0.001
Mean shell length	R = +0.68, p = 0.004	ns
Standardized Richness (n = 15)	ns	R = +0.81, *p* = 0.0001
Dominance	ns	R = −0.76, *p* = 0.0007
Shannon (H)	ns	R = +0.78, *p* = 0.0004
Too fragmented to be certain	ns	R = −0.76, *p* = 0.0007
Salinity	ns	R = +0.76, *p* = 0.0006
nMDS1	ns	—
nMDS2	ns	ns

The absence of correlation between preferred host (*A. segmentum*) abundance and prevalence (Table 1) rules out fluctuating host availability as a limiting factor of trematode distribution^23^. The median shell length of infested valves of *A. segmentum* was significantly larger than that of their non-infested counterparts (Mann-Whitney *U*, *p* = 2.21E-34), likely due to the accumulation of parasites through ontogeny. Prevalence values were positively and significantly correlated with host shell length (r = +0.68, *p* = 0.004**)**, however there were no significant associations between shell length and either flooding pulses or nMDS1 scores (Fig. 2C and Table 1). This suggests that other environmental or ecological factors, acting as drivers of host shell length, were unlikely to have indirectly driven the temporal trend of trematode prevalence. Similarly, the lack of correlative relationships between prevalence and standardized richness, dominance, and Shannon diversity (Table 1) suggests that fluctuating biodiversity did not exhibit direct/linear control over trematode-bivalve interactions.

**Figure 3:**
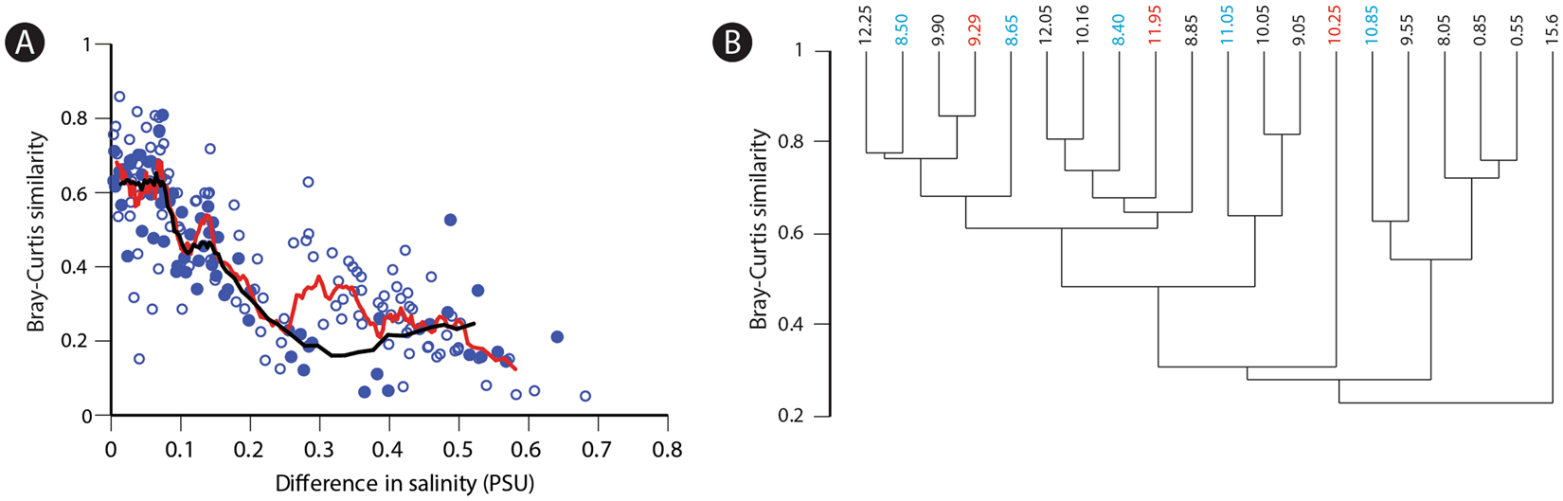
Turnover and ecological similarity of assemblages across core 204-S7. (**A**) Turnover estimated by pairwise comparison of Bray-Curtis similarity indices and environmental distance (nMDS1 salinity). Solid circles indicate pairwise comparisons between lagoonal muddy samples from core depths of 8.50–12.25 meters and the black line indicates the ten point running average. Hollow circles indicate all other comparisons. The red line indicates the ten point running average for all comparisons. (**B**) Q-mode cluster analysis (UPGMA algorithm, Bray-Curtis similarity). Samples with trematode prevalence values of *Abra segmentum* greater than and less than the 95% CI are indicated in red and blue, respectively. Note how the samples of either exceptionally low or high prevalence values are randomly distributed across the dendrogram.

The role of more complex, community-level factors that may have influenced the distribution of trematode parasites can be examined by evaluating the distribution of samples and their constituent taxa in the nMDS space and assessing faunal similarity using Bray-Curtis pairwise comparisons to measure faunal turnover throughout the length of the core (Fig. 3A). Samples retrieved from brackish muds only (8.50–12.25 m core depth) display a comparable amount of turnover to that identified when comparing samples from both freshwater and brackish environments. However, the dendrogram derived from the Q-mode cluster analysis of the samples included in the nMDS ordination demonstrates that samples recording elevated or subdued trematode prevalence were distributed haphazardly across the dendrogram topology. Consequently, community structure/turnover (Fig. 3B) is unlikely to have been a driving factor of trematode prevalence within Holocene lagoonal facies.

Sample nMDS1 scores were negatively correlated with the proportion of *Abra* valves that were too fragmented to evaluate in terms of infestation status. This pattern raises the question of how fragmentation might have influenced the parasite record (i.e., were valves with trematode pits more prone to fragmentation than non-infested valves?). All *Abra* valves were classified as either whole or broken, and the broken valves were further categorized into either “sufficiently complete” or “too fragmented” to determine infestation status. There was no significant difference in trematode prevalence values of whole and “sufficiently complete” broken valves (*Χ* ^*2*^, *p* = 0.16). These results suggest that the proportion of “too fragmented” valves was unlikely to represent an important confounding factor in reconstructing the stratigraphic record of trematode dynamics.

Another potential factor affecting parasite prevalence is the fluctuating availability of habitat-area for trematodes during sea-level cycles. The geologically rapid creation of new habitat during flooding pulses and their subsequent destruction during progradation could exert a first order control on trematode prevalence during high frequency cycles. As sea level continues to rise, some settings will be more strongly influenced than others. For instance, densely populated lowlands, estuarine, and riverine settings would likely display the greatest increase in trematode habitat-area during relative sea level rise as a direct effect of flooding and, indirectly, by the landward rise of the groundwater table^40^. Therefore, we hypothesize that gymnophallid trematode prevalence will be more strongly influenced by the creation of new habitat in brackish and freshwater settings than in shallow marine settings. Though not the direct topic of research here, an increase in wetlands created by sea level rise would generate new habitat for the gastropod intermediate hosts of *Schistosoma* ^41^, the trematodes responsible for schistosomiasis in humans.

The fossil record of the northern Adriatic points to a significant association between the prevalence of heterocious parasites and flooding events recording repeated climate-driven sea level shifts. From this historical perspective we posit that the ongoing anthropogenic warming and sea-level rise should trigger a significant upsurge in gymnophallid trematode prevalence and the expansion of wetland habitats ideal for schistosomatid intermediate hosts. The forecasted changes are expected to suppress the fecundity of common benthic organisms, exert negative impacts on ecosystems, impede ecosystem services, and, eventually, negatively affect human well-being.

## Electronic supplementary material

Online Supplementary Info
