# Supplementary material for: Surges in trematode prevalence linked to centennial-scale flooding events in the Adriatic
Source: Sci Rep. 2017 Jul 18;7:5732. doi: 10.1038/s41598-017-05979-6 (PMC5516012; doi:10.1038/s41598-017-05979-6)
Supplement: Supplementary file 1 — Online Supplementary Info [file 41598_2017_5979_MOESM1_ESM.doc]

Surges in trematode prevalence linked to centennial-scale flooding events in the Adriatic

Daniele Scarponi^1^, Michele Azzarone^1^, Michał Kowalewski^2^, John Warren Huntley^3*^

^1^Dipartimento di Scienze Biologiche, Geologiche e Ambientali, University of Bologna, via Selmi 3, Bologna, I-40126, Italy

^2^Florida Museum of Natural History, University of Florida, 1659 Museum Rd., Gainesville, FL 32611, USA

^3^Department of Geological Sciences, University of Missouri, 101 Geology Building, Columbia, MO 65211

**E-mail:* [*huntleyj@missouri.edu*](mailto:huntleyj@missouri.edu)

**Online Content**

**Methods**

Sixty-one 10cm-thick bulk samples were collected on average every 22 cm from the top 20 meters of core 204-S7 (Fig. 2A), dried at 45˚C, soaked in ~4% H_2_O_2_, and washed through a 1mm sieve (Scarponi and Angeletti, 2008). A total of 45 out of 61 samples contained mollusk fossils (Extended Data Fig. S1). For each of the fossiliferous samples, all complete or unique individual specimens (i.e., bivalve umbo/gastropod apex) were identified to the species level (when possible). The resulting taxon abundance matrix included 3,151 individuals from 26 genera and 31 species (Extended Data Table S1). Of the total matrix abundance 50% (i.e., 1012 out of 2022 valves) were represented by the brackish, deposit-feeding and infaunal bivalve Abra segmentum (Récluz, 1843). All A. segmentum valves were examined with an Askania stereo microscope (at 63x magnification) for the presence of oval- to irregular-shaped pits with raised rims (Fig. 1C), which are indicative of trematode infestation (Ruiz and Lindberg, 1989). Each valve was classified as parasitized, non-parasitized, or too fragmented to be certain. Prevalence was calculated as the number of parasitized valves divided by the total number of valves in a sample. Anterior-posterior length (APL) was measured for all whole valves. For all fragmented valves, APL was estimated based on chondrophore length by reduced major axis regression (RMA). A set of 23 right and 19 left complete valves (Extended Data Fig. S2) was used to relate chondrophore length to APL.

Multivariate ordinations were performed to explore the relationships between faunal composition and environmental gradients. Detrended Correspondence Analyses and non-metric Multidimensional Scaling (nMDS) ordinations returned comparable patterns while varying the taxon and sample thresholds; Extended Data Figs S3A-B; S4). Here we report the results of nMDS in 2-dimensions based on Bray-Curtis distances (metaMDS function; “vegan” package in R) with singleton taxa, monospecific samples, and samples with fewer than 15 specimens excluded from analysis. In the case of bivalves, each valve or unique fragment was counted as a 0.5 specimen (Kowalewski and Bambach, 2000). The final matrix for the nMDS ordination was composed of 20 samples, 17 species, and 2023 specimens (median sample size 75 specimens). Only nMDS axis 1 sample scores, obtained from a log-transformed matrix of specimen raw counts (which consider both shape and size of the count vectors), are reported here (Fig. 2; see Extended Data Fig. S4A-F for more detailed results).

An independent sample-level salinity proxy was calculated via the weighted average of a sub-set of taxa for which optimum salinity values were known (Extended Data Table S2). An RMA regression model between sample-level salinity estimates and nMDS1 scores was calculated so that a salinity proxy could be estimated for samples that were comprised of less than 30% of specimens from taxa for which optimum salinity values were known (Extended Data Table S3; Extended Data Fig. S5).

The extent of faunal turnover among samples along the core was assessed by the pairwise comparison of Bray-Curtis similarity values between samples and salinity estimates used as a proxy of “environmental distance” (Anderson et al., 2011). A Q-mode cluster analysis with paired-group UPGMA algorithm and Bray-Curtis similarity measure was applied to the data matrix utilized in the nMDS to determine the relationship between community structure and trematode prevalence. Finally, Spearman rank correlation analyses were carried out to test for potential interdependence between prevalence estimates and environmental and bio-ecological estimators (nMDS1 scores, shell length, indices of diversity and fragmentation). Randomizations (10,000 iterations each) were conducted for prevalence and shell length estimates to assess the range of values (95% and 99% confidence intervals) expected under the null model of no change through time. The analyses where carried out in R (R Development Core, 2016), PAST v.2.17c (Hammer et al., 2001), and SAS v.9.3 (Der and Everit, 2015).

***Sequence Stratigraphic Framework Of The Latest Quaternary Po Plain Succession***

The latest Quaternary (<30ky) succession of the Po Plain is a few tens of meters thick and extensive subsurface investigations conducted during the last decades have led to a detailed reconstruction of its stratigraphic architecture (e.g., Amorosi et al., 2005; Scarponi and Angeletti, 2008; Amorosi et al., 2014; Scarponi et al., submitted). This genetically related, wedge-shaped package of strata represents the Last Glacial Maximum (LGM) depositional sequence. The laterally extensive fluvial-channel and associated floodplain deposits of the lower part of the succession are interpreted to record progradation and aggradation during the LGM and early sea-level rise (30-14ky). LGM deposit are overlaid by a few meters of rapidly retrogradational stack of nearshore (barrier-lagoon-estuary system) to shallow marine fossil rich mudstones, in part filling the coastal accommodation created during rapid sea-level rise (14-7ky). Ensuing aggradation followed by progradation into the Adriatic Basin represents a distinctive stacking pattern generated by increasingly strong riverine sedimentation enhanced by the middle-late Holocene deceleration of sea-level rise (>6ky). Hence, in distal locations, the coastal accommodation created by the synergistic influence of eustasy, compaction, and subsidence during the early to middle Holocene was filled by a tract of shallowing upward marine and progradational deltaic facies. Whereas, in proximal locations, i.e. the targeted area of this study, the stratigraphic architecture of the LGM depositional sequence is dominated by aggradational alluvial and upper coastal plain facies associations, passing upwards into retrogradational muddy coastal plain to brackish deposits followed by a complex interplay of brackish and subaerial/freshwater (lower delta) plain deposits recording the relative decrease in Holocene sea-level rise.

Recurrent stacking of bedsets and their bounding surfaces have been mapped across shallow marine deposits of the coastal successions targeted here and have been traced landward. Following previous and ongoing investigations, several higher-order depositional cycles defined by their characteristic bounding flooding surfaces, internal stacking patterns, and geometric relations to surrounding strata characterize the internal structure of the LGM sequence and are interpreted here as parasequences (Amorosi et al., 2017). High-resolution chronostratigraphic control indicates that the majority of the parasequences formed on millennial time scales (with durations of about 2000 yrs), and a subset formed on centennial time periods (Scarponi et al., 2013; Amorosi et al., 2017).

Stratigraphic framework of core 204-S7

Core 204-S7, 40 meters-long (Extended Data Fig. S1), was drilled 0.5 m above sea level (Latitude 44.725577º N, Longitude 11.940878º E), south of Ostellato, Italy. The bottom of the core is comprised of two meters thick sedimentary package with sparse freshwater macrofossils and display an overall fining upward tendency (from medium sand to silty-clay), interpreted as fluvial-channel deposit. These coarse grained deposits are capped by eight meters of multi-coloured (yellow to brown), macrofossil-barren, clay to silty-clay (~31 to ~38 m core depth), intercalated by thin sandy layers are interpreted as well-drained floodplain succession deposited during pre-glacial (>32ky) times (Amorosi et al., 2005).

The overlying 31.0-24.7m interval primarily records late glacial dynamics (i.e., lowstand deposition) and is characterized by a ~3m thick fluvial channel body, consisting of amalgamated medium sands with an erosional base resting on top of a stiff calcareous horizon (inceptisol). The sharp erosional surface located at 31m core depth on top of the inceptisol represents the sequence boundary of the Last Glacial Maximum 4th order depositional sequence (Amorosi et al, 2005). Whereas, the upper boundary of this fluvial package records a sharp transition to clayey-silt and silty-clay, in turn overlain by a two meter thick multi-coloured (yellow to brown) deposits characterized by relatively abundant macrofossil (mainly fragments) of land and freshwater mollusks, otherwise showing comparable features to those retrieved between 38-31m core depth. Hence, suggestive of overbank and well-drained floodplain deposits.

Between 24.7 and 20.8m core depth, emplaced on top of underlying muds, a 4m thick, amalgamated and coarse-grained but fining-upward body showing scanty freshwater mollusks is retrieved. This body is interpreted as the sedimentary output of a fluvial channel and the sharp contact delimitating this coarse-grained unit from underlying floodplain deposits is interpreted as the regional transgressive surface (TS) (Bruno et al., 2016). The ~1.0 meter thick homogenous dark brown silty-clay with scanty freshwater mollusks reflect the first record in the core of coastal plain poorly drained deposits and/or temporary standing water bodies. At 19.60m, a sharp facies shift is recorded at the base of a ca. 1.3m thick medium sand with multiple centimeter-scale silt intercalations. This sandy lithosome records the first appearance in the core of scattered specimens of brackish taxa: *Cerastoderma* *glaucum* and *Abra segmentum* (Extended Data Table S1). Hence, the shift of facies from poorly drained floodplain/swamp to brackish settings is retained to represent a flooding surface. Another flooding surface identified by means of micro-sedimentary inferences (Amorosi et al., 2017), is located at 16.20m core depth, on top of a thin peat layer. The ~2m thick dark organic rich clay with silty intercalations recording freshwater gastropod (hydrobiids and *Teodoxus fluviatilis*) bracketed between coarse sand and the peat layer, records the rapid transition from a brackish setting to a poorly drained floodplain/swamp. The overlying ~4m thick grey clay interval (16.20 to 12.25m core depth) bearing scanty macrofossils records, based on micro-sedimentary inferences, a further parasequence boundary around 14.30m core depth. This flooding surface separates swamp deposits below from brackish to swamp deposits above.

The interval 12.60-7.70m core depth is characterized by fine to medium decimetric to metric sand bodies alternating with clay-silt organic rich deposits organized into stacking patterns of meter scale, coarsening-upward, of back barrier facies. This staking pattern and the associated stock of brackish species that tend to decrease in abundance from fine to coarse deposits represents the set of three small scale cycles thoroughly investigated in this study. The uppermost one is capped by grey, fine to medium sand fluvial package, 1.0m thick with internal fining-upward trends. This body is interpreted to reflect sand inputs into the lagoon by fluvial channels and it progressively infill. Between 6 and 1.5m core depth is characterized by an overall aggradational and cyclic staking pattern of muddy to sandy deposits with scattered freshwater and or pulmonate molluscs. Based on microfaunal and sedimentary inferences this interval records the repeated alternation of swamp, floodplain and overbank deposits ranging from one to few meter thick and recording the alternating development coastal plain dynamics related mainly to autogenic Po Delta dynamics. Within this interval three flooding surfaces delimiting millennial scale cycles are recognized at 6.2, ~5.0 and ~3.0m core depth. A last flooding surface at 1.5m core depth is clearly recorded by a sharp transition from poorly drained floodplain muds and silty-muds recording the brackish bivalve *Abra segmentum* representing the last developments of short lived lagoon.

**Biofacies-Refined Interpretation of Sequence Stratigraphic Architecture**

The vertical trend of nMDS1 sample scores and their strong correlation with salinity, a common ecological driver in back barrier settings (see Amorosi et al., 2014), further demonstrates the utility of macrofossil data to augment stratigraphic interpretation in cored deposits.

At the scale of the overall sedimentary package examined (Fig. 2), the nMDS-derived salinity pattern supports the control of glacio-eustatic forcing on the development of the Late Glacial Depositional sequence. Specifically, the lower part (i.e., 20 to 10.30m in Fig. 2) of the v-shaped nMDS-derived salinity profile records the increasing influence of the sea (supported by the overall increase in salinity), which is consistent with a retrogradational stacking pattern of back barrier facies (i.e., floodplain to lagoon settings). Whereas the upper shift toward lower salinity values, recorded after 6 ky BP (see Amorosi et al. 2017), is consistent with progradational to aggradational stacking patterns of brackish, freshwater and poorly drained floodplain deposits of the regressive systems tract (Figs 1b-2a,b).

At a higher resolution, the stratigraphic trajectory of nMDS1 sample scores highlights five flooding pulses depicted by major increases in salinity (here interpreted as parasequence bounding surfaces), followed by a gradual return to previous conditions (see Fig. 2). These abrupt contacts (e.g., ~12‰ salinity shift at 9.5m core depth in Fig. 2), are interpreted to represent non-Waltherian facies dislocations, with the overlying facies recording increased marine influence rather than a simple lateral shift to an adjacent environment relative to the underlying facies.

The three salinity shifts recognized at 15.5, 12.3, and 1.1m core depth (Fig. 2) represent parasequence bounding surfaces developed over millennial time scales. Whereas the remaining two are interpreted as higher frequency (centennial) pulses representing short lived, conditions of these back barrier settings. Accordingly, it is possible to subdivide parasequence 4, developed at the time of maximum marine ingression (Fig. 2B), into a set of three higher frequency (sub-millennial scale) units bounded by significant (i.e., non Waltherian) shifts of facies indicating rapid transition from mesohaline to polyhaline dominated paleoenvironments (Fig. 2B).

Extended Data Table S1—Mollusc dataset: sixty-five bulk samples (10cm thick), collected at a vertical spacing of no more than 50 cm from core 204-S7 drilled ~24km west of the modern shoreline. Nineteen of the 65 samples were barren, the remaining 46 samples yielded 3171 individuals from 26 genera and 32 species. Note: Ecrobia groups a stock of taxa very similar (Ecrobia and Hydrobia), mainly distinguishable by means of soft tissues.

| **Family** | **Well depth (m)** | **24.05** | **23.05** | **22.05** | **21.05** | **20.05** | **19.75** | **19.5** | **19.05** | **18.95** |
| --- | --- | --- | --- | --- | --- | --- | --- | --- | --- | --- |
|  | **Genus/Species** |  |  |  |  |  |  |  |  |  |
| SEMELIDAE | *Abra segmentum* | 0 | 0 | 0 | 0 | 0 | 0 | 0 | 1 | *4* |
| SEMELIDAE | *Abra* sp. | 0 | 0 | 0 | 0 | 0 | 0 | 0 | 0 | 0 |
| CARDIIDAE | *Cerastoderma glaucum* | 0 | 0 | 0 | 0 | 0 | 0 | ***1*** | 1 | 1 |
| CORBULIDAE | *Lentidium mediterraneum* | 0 | 0 | 0 | 0 | 0 | 0 | 0 | 0 | 0 |
| LUCINIDAE | *Loripes orbiculatus* | 0 | 0 | 0 | 0 | 1 | 0 | 0 | 2 | 0 |
| MYTILIDAE | *Modiolus* sp. | 0 | 0 | 0 | 0 | 0 | 0 | 0 | 0 | 0 |
| MONTACUTIDAE | *Kurtiella bidentata* | 0 | 0 | 0 | 0 | 0 | 0 | 0 | 0 | 0 |
| SPHAERIDAE | *Pisidium amnicum*_cf | 1 | 0 | 0 | 0 | 0 | 0 | 0 | 0 | 0 |
| SPHAERIDAE | *Pisidium subtruncatum*_cf | 0 | 0 | 0 | 0 | 2 | 0 | 0 | 0 | 0 |
| VENERIDAE | *Polititapes aureus* | 0 | 0 | 0 | 0 | 0 | 0 | 0 | 0 | 0 |
| VENERIDAE | *Polititapes* sp. | 0 | 0 | 0 | 0 | 0 | 0 | 0 | 0 | 0 |
| MACTRIDAE | *Spisula* sp. | 0 | 0 | 0 | 0 | 0 | 0 | 0 | 0 | 0 |
| PLANORBIDAE | *Anisus leucostoma* | 0 | 0 | 3 | 0 | 0 | 0 | 0 | 0 | 0 |
| PLANORBIDAE | *Anisus vortex* | 0 | 0 | 1 | 0 | 0 | 0 | 0 | 0 | 0 |
| BITHYNIIDAE | *Bithynia tentaculata*_cf | 2 | 0 | 8 | 0 | 0 | 0 | 0 | 0 | 0 |
| Cerithiopsidae | *Certithiopsis* sp. | 0 | 0 | 0 | 0 | 0 | 0 | 0 | 0 | 0 |
| CERITHIIDAE | *Bittium reticulatum* | 0 | 0 | 0 | 0 | 0 | 0 | 0 | 1 | *1* |
| CERITHIIDAE | *Cerithium vulgatum* | 0 | 0 | 0 | 0 | 0 | 0 | 0 | 0 | 0 |
| CERITHIIDAE | *Cerithium* sp. | 0 | 0 | 0 | 0 | 0 | 0 | 0 | 0 | 0 |
| PYRAMIDELLIDAE | *Chrysallida interstincta* | 0 | 0 | 0 | 0 | 0 | 0 | 0 | 0 | 0 |
| PYRAMIDELLIDAE | *Chrysallida* sp.1 | 0 | 0 | 0 | 0 | 0 | 0 | 0 | 0 | 0 |
| HYDROBIIDAE | *Ecrobia ventrosa_gr.* | 0 | 0 | 0 | 0 | 0 | 0 | 2 | 1 | 2 |
| NASSARIIDAE | *Nassarius nitidus* | 0 | 0 | 0 | 0 | 0 | 0 | 0 | 0 | 0 |
| NASSARIIDAE | *Nassarius* sp. | 0 | 0 | 0 | 0 | 0 | 0 | 0 | 0 | 0 |
| SUCCINEIDAE | *Oxyloma* sp. | 0 | 0 | 0 | 0 | 0 | 0 | 0 | 0 | 0 |
| RISSOIDAE | *Pusillina lineolata* | 0 | 0 | 0 | 0 | 0 | 0 | 0 | 0 | 0 |
| RISSOIDAE | *Pusillina marginata* | 0 | 0 | 0 | 0 | 0 | 0 | 0 | 0 | 0 |
| RISSOIDAE | *Pusillina* sp. | 0 | 0 | 0 | 0 | 0 | 0 | 0 | 0 | 0 |
| RETUSIDAE | *Retusa mammillata* | 0 | 0 | 0 | 0 | 0 | 0 | 0 | 0 | 0 |
| RISSOIDAE | *Rissoa ventricosa* | 0 | 0 | 0 | 0 | 0 | 0 | 0 | 0 | 0 |
| RISSOIDAE | *Rissoa membranacea* | 0 | 0 | 0 | 0 | 0 | 0 | 0 | 0 | 0 |
| RISSOIDAE | *Rissoa* sp. | 0 | 0 | 0 | 0 | 0 | 0 | 0 | 0 | 0 |
| RISSOIDAE | *indet* | 0 | 0 | 0 | 0 | 0 | 0 | 0 | 0 | 0 |
| NERITIDAE | *Smaragdia viridis* | 0 | 0 | 0 | 0 | 0 | 0 | 0 | 0 | 0 |
| SUCCINEIDAE | *Succinella oblonga* | 0 | 0 | 2 | 0 | 0 | 0 | 0 | 0 | 0 |
| NERITIDAE | *Theodoxus fluviatilis* | 0 | 0 | 0 | 0 | 0 | 0 | 0 | 0 | 0 |
| PYRAMIDELLIDAE | *Turbonilla lactea* | 0 | 0 | 0 | 0 | 0 | 0 | 0 | 0 | 0 |
| VALVATIDAE | *Valvata piscinalis* | 2 | 0 | 2 | 0 | 0 | 2 | 0 | 0 | 0 |
| **sample abundance** | | **5** | **0** | **16** | **0** | **3** | **2** | **3** | **6** | **8** |

| **18.55** | **18.05** | **17.55** | **17.05** | **16.55** | **16.05** | **15.9** | **15.6** | **15.15** | **14.35** | **14.05** | **13.5** | **13.4** | **13.05** | **12.98** |
| --- | --- | --- | --- | --- | --- | --- | --- | --- | --- | --- | --- | --- | --- | --- |
|  |  |  |  |  |  |  |  |  |  |  |  |  |  |  |
| 1 | 0 | 0 | 0 | 0 | 0 | 0 | 0 | 1 | 0 | 0 | 0 | 0 | 0 | 0 |
| 0 | 0 | 0 | 0 | 0 | 0 | 0 | 0 | 0 | 2 | 0 | 0 | 0 | 0 | 0 |
| 0 | 0 | 0 | 0 | 0 | 0 | *1* | *1* | 0 | 0 | 0 | 0 | 0 | 0 | 0 |
| 0 | 0 | 0 | 0 | 0 | 0 | 0 | 0 | 0 | 0 | 0 | 0 | 0 | 0 | 0 |
| 0 | 0 | 0 | 0 | 0 | 0 | 0 | 0 | 0 | 0 | 0 | 0 | 0 | 0 | 0 |
| 0 | 0 | 0 | 0 | 0 | 0 | 0 | 0 | 0 | 0 | 0 | 0 | 0 | 0 | 0 |
| 0 | 0 | 0 | 0 | 0 | 0 | 0 | 0 | 0 | 0 | 0 | 0 | 0 | 0 | 0 |
| 0 | 0 | 0 | 0 | 0 | 0 | 0 | 0 | 0 | 0 | 0 | 0 | 0 | 0 | 0 |
| 0 | 0 | 0 | 0 | 0 | 0 | 0 | 0 | 0 | 0 | 0 | 0 | 0 | 0 | 0 |
| 0 | 0 | 0 | 0 | 0 | 0 | 0 | 0 | 0 | 0 | 0 | 0 | 0 | 0 | 0 |
| 0 | 0 | 0 | 0 | 0 | 0 | 0 | 0 | 0 | 0 | 0 | 0 | 0 | 0 | 0 |
| 0 | 0 | 0 | 0 | 0 | 0 | 0 | 0 | 0 | 0 | 0 | 0 | 0 | 0 | 0 |
| 0 | 0 | 0 | 0 | 0 | 0 | 0 | 0 | 0 | 0 | 0 | 0 | 0 | 0 | 0 |
| 0 | 0 | 0 | 0 | 0 | 0 | 0 | 0 | 0 | 0 | 0 | 0 | 0 | 0 | 0 |
| 0 | 0 | 18 | 0 | 0 | 0 | 0 | 0 | 0 | 0 | 0 | 0 | 0 | 0 | 3 |
| 0 | 0 | 0 | 0 | 0 | 0 | 0 | 0 | 0 | 0 | 0 | 0 | 0 | 0 | 0 |
| 0 | 0 | 0 | 0 | 0 | 0 | 0 | 0 | 0 | 0 | 0 | 0 | 0 | 0 | 1 |
| 0 | 0 | 0 | 0 | 0 | 0 | 0 | 0 | 0 | 0 | 0 | 0 | 0 | 0 | 0 |
| 0 | 0 | 0 | 0 | 0 | 0 | 0 | 0 | 0 | 0 | 0 | 0 | 0 | 0 | 0 |
| 0 | 0 | 0 | 0 | 0 | 0 | 0 | 0 | 0 | 0 | 0 | 0 | 0 | 0 | 0 |
| 0 | 0 | 0 | 0 | 0 | 0 | 0 | 0 | 0 | 0 | 0 | 0 | 0 | 0 | 0 |
| 0 | 0 | 0 | 0 | 0 | 0 | 10 | 61 | 0 | 0 | 0 | 1 | 0 | 0 | 0 |
| 0 | 0 | 0 | 0 | 0 | 0 | 0 | 0 | 0 | 0 | 0 | 0 | 0 | 0 | 0 |
| 0 | 0 | 0 | 0 | 0 | 0 | 0 | 0 | 0 | 0 | 0 | 0 | 0 | 0 | 0 |
| 0 | 0 | 0 | 0 | 0 | 0 | 0 | 0 | 0 | 0 | 0 | 0 | 0 | 0 | 0 |
| 0 | 0 | 0 | 0 | 0 | 0 | 0 | 0 | 0 | 0 | 0 | 0 | 0 | 0 | 0 |
| 0 | 0 | 0 | 0 | 0 | 0 | 0 | 0 | 0 | 0 | 0 | 0 | 0 | 0 | 0 |
| 0 | 0 | 0 | 0 | 0 | 0 | 0 | 0 | 0 | 0 | 0 | 0 | 0 | 0 | 0 |
| 0 | 0 | 0 | 0 | 0 | 0 | 0 | 0 | 0 | 0 | 0 | 0 | 0 | 0 | 0 |
| 0 | 0 | 0 | 0 | 0 | 0 | 0 | 0 | 0 | 0 | 0 | 0 | 0 | 0 | 0 |
| 0 | 0 | 0 | 0 | 0 | 0 | 0 | 0 | 0 | 0 | 0 | 0 | 0 | 0 | 0 |
| 0 | 0 | 0 | 0 | 0 | 0 | 0 | 0 | 0 | 0 | 0 | 0 | 0 | 0 | 0 |
| 0 | 0 | 0 | 0 | 0 | 0 | 0 | 0 | 0 | 0 | 0 | 0 | 0 | 0 | 0 |
| 0 | 0 | 0 | 0 | 0 | 1 | 0 | 0 | 0 | 0 | 0 | 0 | 0 | 0 | 0 |
| 0 | 0 | 0 | 0 | 0 | 0 | 0 | 0 | 0 | 0 | 0 | 0 | 0 | 0 | 0 |
| 0 | 0 | 0 | 0 | 0 | 0 | 4 | 0 | 1 | 0 | 0 | 0 | 0 | 0 | 0 |
| 0 | 0 | 0 | 0 | 0 | 0 | 0 | 0 | 0 | 0 | 0 | 0 | 0 | 0 | 0 |
| 0 | 0 | 0 | 0 | 0 | 0 | 0 | 0 | 0 | 0 | 0 | 0 | 0 | 0 | 0 |
| **1** | **0** | **18** | **0** | **0** | **1** | **15** | **62** | **2** | **2** | **0** | **1** | **0** | **0** | **4** |

| **12.55** | **12.45** | **12.25** | **12.05** | **11.95** | **11.55** | **11.35** | **11.05** | **10.85** | **10.55** | **10.25** | **10.16** | **10.05** | **9.9** | **9.55** |
| --- | --- | --- | --- | --- | --- | --- | --- | --- | --- | --- | --- | --- | --- | --- |
|  |  |  |  |  |  |  |  |  |  |  |  |  |  |  |
| 2 | 1 | 35 | 98 | 50 | 8 | 1 | 33 | 14 | 0 | 218 | 80 | 21 | 51 | 16 |
| 0 | 0 | 0 | 0 | 0 | 0 | 0 | 0 | 0 | 0 | 0 | 0 | 0 | 0 | 0 |
| 0 | 2 | 10 | 11 | 15 | 0 | 1 | 9 | 2 | 0 | 44 | 28 | 7 | 12 | 0 |
| 0 | 0 | 0 | 0 | 0 | 0 | 0 | 0 | 0 | 0 | 3 | 0 | 0 | 0 | 0 |
| 0 | 1 | 30 | 67 | 46 | 2 | 3 | 11 | 3 | 0 | 240 | 73 | 19 | 31 | 0 |
| 0 | 0 | 1 | 0 | 0 | 0 | 0 | 0 | 0 | 0 | 3 | 2 | 0 | 0 | 0 |
| 0 | 0 | 0 | 1 | 0 | 0 | 0 | 0 | 0 | 0 | 0 | 0 | 0 | 0 | 0 |
| 0 | 0 | 0 | 0 | 0 | 0 | 0 | 0 | 0 | 0 | 0 | 0 | 0 | 0 | 0 |
| 0 | 0 | 0 | 0 | 0 | 0 | 0 | 0 | 0 | 0 | 0 | 0 | 0 | 0 | 0 |
| 0 | 0 | 0 | 0 | 0 | 0 | 0 | 0 | 0 | 0 | 0 | 0 | 0 | 0 | 0 |
| 0 | 0 | 0 | 0 | 0 | 0 | 0 | 0 | 0 | 0 | **3** | 1 | 0 | 1 | 0 |
| 0 | 0 | 0 | 0 | 0 | 0 | 0 | 0 | 0 | 0 | 0 | 1 | 0 | 0 | 0 |
| 0 | 0 | 0 | 0 | 0 | 0 | 0 | 0 | 0 | 0 | 0 | 0 | 0 | 0 | 0 |
| 0 | 0 | 0 | 0 | 0 | 0 | 0 | 0 | 0 | 0 | 0 | 0 | 0 | 0 | 0 |
| 0 | 0 | 0 | 0 | 0 | 2 | 0 | 0 | 0 | 0 | 0 | 0 | 1 | 0 | 3 |
| 0 | 0 | 0 | 0 | 0 | 0 | 0 | 0 | 0 | 0 | 2 | 0 | 0 | 0 | 0 |
| 0 | 0 | 6 | 27 | 45 | 3 | 2 | 3 | 4 | 0 | 118 | 33 | 14 | 24 | 0 |
| 0 | 0 | 0 | 0 | 0 | 0 | 0 | 0 | 0 | 0 | 0 | 0 | 0 | 0 | 0 |
| 0 | 0 | 0 | 1 | 0 | 0 | 0 | 0 | 0 | 0 | 0 | 1 | 0 | 0 | 0 |
| 0 | 0 | 0 | 0 | 0 | 0 | 0 | 0 | 0 | 0 | 3 | 2 | 0 | 3 | 0 |
| 0 | 0 | 0 | 0 | 0 | 0 | 0 | 0 | 0 | 0 | 2 | 0 | 0 | 0 | 0 |
| 0 | 0 | 16 | 24 | 59 | 2 | 6 | 2 | 4 | 0 | 120 | 17 | 5 | 18 | 6 |
| 0 | 0 | 0 | 0 | 0 | 0 | 0 | 0 | 0 | 0 | 1 | 0 | 0 | 0 | 0 |
| 0 | 0 | 0 | 0 | 0 | 0 | 0 | 0 | 0 | 0 | 1 | 0 | 0 | 0 | 0 |
| 0 | 0 | 0 | 0 | 0 | 0 | 0 | 0 | 0 | 0 | 0 | 1 | 0 | 0 | 0 |
| 0 | 0 | 7 | 0 | 4 | 0 | 0 | 0 | 0 | 0 | 20 | 0 | 0 | 0 | 0 |
| 0 | 0 | 0 | 1 | 0 | 0 | 0 | 0 | 0 | 0 | 0 | 0 | 0 | 0 | 0 |
| 0 | 0 | 0 | 0 | 0 | 0 | 0 | 0 | 0 | 0 | 0 | 0 | 0 | 2 | 0 |
| 0 | 0 | 0 | 1 | 1 | 0 | 0 | 1 | 0 | 0 | 7 | 1 | 0 | 0 | 0 |
| 0 | 0 | 0 | 0 | 0 | 0 | 0 | 0 | 0 | 0 | 0 | 0 | 0 | 0 | 0 |
| 0 | 0 | 0 | 0 | 1 | 2 | 0 | 0 | 0 | 0 | 2 | 12 | 1 | 3 | 0 |
| 0 | 0 | 0 | 0 | 6 | 0 | 0 | 0 | 1 | 0 | 0 | 0 | 1 | 0 | 0 |
| 0 | 0 | 0 | 3 | 0 | 0 | 0 | 0 | 0 | 0 | 0 | 0 | 0 | 0 | 0 |
| 0 | 0 | 0 | 0 | 0 | 0 | 0 | 0 | 0 | 0 | 0 | 0 | 0 | 0 | 0 |
| 0 | 0 | 0 | 0 | 0 | 0 | 0 | 0 | 0 | 0 | 0 | 0 | 0 | 0 | 0 |
| 0 | 0 | 0 | 0 | 0 | 0 | 0 | 0 | 0 | 0 | 0 | 0 | 0 | 0 | 0 |
| 0 | 0 | 0 | 0 | 0 | 0 | 0 | 0 | 0 | 0 | 0 | 0 | 1 | 0 | 0 |
| 0 | 1 | 0 | 0 | 0 | 0 | 0 | 0 | 0 | 0 | 0 | 0 | 0 | 0 | 0 |
| **2** | **5** | **105** | **234** | **227** | **19** | **13** | **59** | **28** | **0** | **787** | **252** | **70** | **145** | **25** |

| **9.27** | **9.05** | **8.95** | **8.85** | **8.65** | **8.5** | **8.4** | **8.05** | **7.85** | **7.55** | **7.05** | **6.55** | **6.05** | **5.95** | **5.45** |
| --- | --- | --- | --- | --- | --- | --- | --- | --- | --- | --- | --- | --- | --- | --- |
|  |  |  |  |  |  |  |  |  |  |  |  |  |  |  |
| 44 | 18 | 1 | 47 | 59 | 124 | 54 | 4 | 0 | 0 | 1 | 0 | 0 | 0 | 0 |
| 0 | 0 | 0 | 0 | 1 | 0 | 0 | 0 | 0 | 0 | 0 | 0 | 0 | 0 | 0 |
| 5 | 6 | 0 | 34 | 31 | 29 | 18 | 0 | 0 | 0 | 0 | 0 | 0 | 0 | 0 |
| 0 | 0 | 0 | 1 | 1 | 0 | 0 | 0 | 0 | 0 | 0 | 0 | 0 | 0 | 0 |
| 39 | 28 | 0 | 23 | 16 | 30 | 29 | 3 | 0 | 0 | 0 | 0 | 0 | 0 | 0 |
| 0 | 0 | 0 | 0 | 1 | 0 | 0 | 0 | 0 | 0 | 0 | 0 | 0 | 0 | 0 |
| 0 | 0 | 0 | 0 | 0 | 0 | 0 | 0 | 0 | 0 | 0 | 0 | 0 | 0 | 0 |
| 0 | 0 | 0 | 0 | 0 | 0 | 0 | 0 | 0 | 0 | 0 | 0 | 0 | 0 | 0 |
| 0 | 0 | 0 | 0 | 0 | 0 | 0 | 0 | 0 | 0 | 0 | 0 | 0 | 0 | 0 |
| 0 | 0 | 0 | 3 | 4 | 3 | 0 | 0 | 0 | 0 | 0 | 0 | 0 | 0 | 0 |
| 0 | 0 | 0 | 0 | 0 | 0 | 0 | 0 | 0 | 0 | 0 | 0 | 0 | 0 | 0 |
| 0 | 0 | 0 | 0 | 0 | 0 | 0 | 0 | 0 | 0 | 0 | 0 | 0 | 0 | 0 |
| 0 | 0 | 0 | 0 | 0 | 0 | 0 | 0 | 0 | 0 | 0 | 0 | 0 | 0 | 0 |
| 0 | 0 | 0 | 0 | 0 | 0 | 0 | 0 | 0 | 0 | 0 | 0 | 0 | 0 | 0 |
| 0 | 0 | 0 | 0 | 0 | 0 | 0 | 0 | 0 | 0 | 0 | 0 | 0 | 0 | 0 |
| 0 | 0 | 0 | 0 | 0 | 0 | 0 | 0 | 0 | 0 | 0 | 0 | 0 | 0 | 0 |
| 19 | 10 | 6 | 63 | 3 | 31 | 4 | 0 | 0 | 0 | 0 | 0 | 0 | 0 | 0 |
| 0 | 0 | 1 | 0 | 1 | 0 | 2 | 0 | 0 | 0 | 0 | 0 | 0 | 0 | 0 |
| 0 | 0 | 0 | 0 | 0 | 0 | 0 | 0 | 0 | 0 | 0 | 0 | 0 | 0 | 0 |
| 0 | 0 | 0 | 1 | 0 | 0 | 2 | 0 | 0 | 0 | 0 | 0 | 0 | 0 | 0 |
| 0 | 0 | 0 | 0 | 0 | 0 | 0 | 0 | 0 | 0 | 0 | 0 | 0 | 0 | 0 |
| 17 | 3 | 0 | 16 | 16 | 39 | 22 | 10 | 8 | 0 | 1 | 0 | 0 | 1 | 0 |
| 0 | 0 | 0 | 0 | 1 | 0 | 0 | 0 | 0 | 0 | 0 | 0 | 0 | 0 | 0 |
| 0 | 0 | 0 | 0 | 0 | 0 | 0 | 0 | 0 | 0 | 0 | 0 | 0 | 0 | 0 |
| 0 | 0 | 0 | 0 | 0 | 0 | 0 | 0 | 0 | 0 | 0 | 0 | 0 | 0 | 0 |
| 5 | 0 | 0 | 0 | 1 | 0 | 0 | 0 | 0 | 0 | 0 | 0 | 0 | 0 | 0 |
| 0 | 0 | 4 | 0 | 0 | 0 | 0 | 0 | 0 | 0 | 0 | 0 | 0 | 0 | 0 |
| 0 | 0 | 0 | 1 | 0 | 0 | 0 | 0 | 0 | 0 | 0 | 0 | 0 | 0 | 0 |
| 0 | 1 | 1 | 6 | 1 | 9 | 0 | 0 | 0 | 0 | 0 | 0 | 0 | 0 | 0 |
| 0 | 0 | 0 | 5 | 3 | 0 | 0 | 0 | 0 | 0 | 0 | 0 | 0 | 0 | 0 |
| 2 | 2 | 0 | 0 | 0 | 3 | 0 | 0 | 0 | 0 | 0 | 0 | 0 | 0 | 0 |
| 0 | 0 | 0 | 4 | 0 | 0 | 0 | 0 | 0 | 0 | 0 | 0 | 0 | 0 | 0 |
| 0 | 0 | 0 | 0 | 0 | 0 | 0 | 0 | 0 | 0 | 0 | 0 | 0 | 0 | 0 |
| 0 | 0 | 0 | 0 | 0 | 0 | 0 | 0 | 0 | 0 | 0 | 0 | 0 | 0 | 0 |
| 0 | 0 | 0 | 0 | 0 | 0 | 0 | 0 | 0 | 0 | 0 | 0 | 0 | 0 | 0 |
| 0 | 0 | 0 | 0 | 0 | 0 | 0 | 0 | 0 | 0 | 0 | 0 | 0 | 0 | 0 |
| 0 | 0 | 0 | 0 | 0 | 0 | 0 | 0 | 0 | 0 | 0 | 0 | 0 | 0 | 0 |
| 0 | 0 | 0 | 0 | 0 | 0 | 0 | 0 | 0 | 0 | 0 | 0 | 0 | 0 | 0 |
| **131** | **68** | **13** | **204** | **139** | **268** | **131** | **17** | **8** | **0** | **2** | **0** | **0** | **1** | **0** |

| **5.05** | **4.55** | **4.05** | **3..45** | **3.05** | **2.55** | **2.05** | **1.59** | **0.96** | **0.85** | **0.55** | **species** |
| --- | --- | --- | --- | --- | --- | --- | --- | --- | --- | --- | --- |
|  |  |  |  |  |  |  |  |  |  |  | **abundance** |
| 0 | 0 | 0 | 0 | 0 | 0 | 0 | 0 | 3 | 17 | 5 | **1012** |
| 0 | 0 | 0 | 0 | 0 | 0 | 0 | 0 | 0 | 0 | 0 | **3** |
| 0 | 0 | 0 | 0 | 0 | 0 | 0 | 0 | 2 | 3 | 4 | **278** |
| 0 | 0 | 0 | 0 | 0 | 0 | 0 | 0 | 0 | 0 | 2 | **7** |
| 0 | 0 | 0 | 0 | 0 | 0 | 0 | 0 | 0 | 0 | 0 | **697** |
| 0 | 0 | 0 | 0 | 0 | 0 | 0 | 0 | 0 | 0 | 0 | **7** |
| 0 | 0 | 0 | 0 | 0 | 0 | 0 | 0 | 0 | 0 | 0 | **1** |
| 0 | 0 | 0 | 0 | 0 | 0 | 0 | 0 | 0 | 0 | 0 | **1** |
| 0 | 0 | 0 | 0 | 0 | 0 | 0 | 0 | 0 | 0 | 0 | **2** |
| 0 | 0 | 0 | 0 | 0 | 0 | 0 | 0 | 0 | 0 | 0 | **10** |
| 0 | 0 | 0 | 0 | 0 | 0 | 0 | 0 | 0 | 0 | 0 | **5** |
| 0 | 0 | 0 | 0 | 0 | 0 | 0 | 0 | 0 | 0 | 0 | **1** |
| 0 | 0 | 0 | 0 | 0 | 0 | 0 | 0 | 0 | 0 | 0 | **3** |
| 0 | 0 | 0 | 0 | 0 | 0 | 0 | 0 | 0 | 0 | 0 | **1** |
| 0 | 1 | 1 | 0 | 0 | 0 | 1 | 0 | 0 | 0 | 1 | **41** |
| 0 | 0 | 0 | 0 | 0 | 0 | 0 | 0 | 0 | 0 | 0 | **2** |
| 0 | 0 | 0 | 0 | 0 | 0 | 0 | 0 | 0 | 0 | 0 | **418** |
| 0 | 0 | 0 | 0 | 0 | 0 | 0 | 0 | 0 | 0 | 0 | **4** |
| 0 | 0 | 0 | 0 | 0 | 0 | 0 | 0 | 0 | 0 | 0 | **2** |
| 0 | 0 | 0 | 0 | 0 | 0 | 0 | 0 | 0 | 0 | 0 | **11** |
| 0 | 0 | 0 | 0 | 0 | 0 | 0 | 0 | 0 | 0 | 0 | **2** |
| 0 | 0 | 0 | 0 | 0 | 0 | 0 | 0 | 1 | 11 | 12 | **513** |
| 0 | 0 | 0 | 0 | 0 | 0 | 0 | 0 | 0 | 0 | 0 | **2** |
| 0 | 0 | 0 | 0 | 0 | 0 | 0 | 0 | 0 | 0 | 0 | **1** |
| 0 | 0 | 0 | 0 | 0 | 0 | 0 | 0 | 0 | 0 | 0 | **1** |
| 0 | 0 | 0 | 0 | 0 | 0 | 0 | 0 | 0 | 0 | 0 | **37** |
| 0 | 0 | 0 | 0 | 0 | 0 | 0 | 0 | 0 | 0 | 1 | **6** |
| 0 | 0 | 0 | 0 | 0 | 0 | 0 | 0 | 0 | 0 | 0 | **3** |
| 0 | 0 | 0 | 0 | 0 | 0 | 0 | 0 | 0 | 0 | 0 | **29** |
| 0 | 0 | 0 | 0 | 0 | 0 | 0 | 0 | 0 | 0 | 0 | **8** |
| 0 | 0 | 0 | 0 | 0 | 0 | 0 | 0 | 0 | 0 | 0 | **28** |
| 0 | 0 | 0 | 0 | 0 | 0 | 0 | 0 | 0 | 0 | 0 | **12** |
| 0 | 0 | 0 | 0 | 0 | 0 | 0 | 0 | 3 | 0 | 0 | **6** |
| 0 | 0 | 0 | 0 | 0 | 0 | 0 | 0 | 0 | 0 | 0 | **1** |
| 0 | 0 | 0 | 0 | 0 | 0 | 0 | 0 | 0 | 0 | 0 | **2** |
| 0 | 0 | 0 | 0 | 0 | 0 | 0 | 0 | 0 | 0 | 0 | **5** |
| 0 | 0 | 0 | 0 | 0 | 0 | 0 | 0 | 0 | 0 | 0 | **1** |
| 0 | 0 | 0 | 0 | 0 | 0 | 1 | 0 | 0 | 0 | 1 | **5** |
| **0** | **1** | **1** | **0** | **0** | **0** | **2** | **0** | **9** | **31** | **26** |  |

**Extended Data Table S2**—Summary of present-day salinity data used for environmental calibration of the mollusc nMDS axis 1 sample scores (nMDS1). The seven species used here were selected based on their abundance in 204-S7 core dataset (Extended Data Table S1) and to ensure continuous coverage along nMDS1. Preferred salinity of species (optima) and salinity range (min - max) were obtained from literature data (References). Midpoint is referred (if available) to the preferred salinity range (optima) of a targeted species.

|  |  | | |  |  |  | |  |  |  |
| --- | --- | --- | --- | --- | --- | --- | --- | --- | --- | --- |
| Taxon | | Salinity (psu) | | | | | References | | | |
|  | | min | max | | optimal | midpoint |  | | | |
| *Loripes orbiculatus* | | 7 | >35 | | 20-35 | 27.5 | Anistratenko 2011; Encyclopedia of Life, 2016; Cilenti et al., 2008 | | | |
| *Cerastoderma glaucum* | | 3 | 61 | | 5.0-38.0 | 21.5 | Gontikaki et al., 2003; Boyden and Russel, 1972 | | | |
| *Bithynia tentaculata* | | 0 | 6.3 | | 0-4.2 | 2.1 | Berezina, 2003 | | | |
| *Abra segmentum* | | 3 | >35 (41) | | 9.0-12.0 | 10.5 | CSB Database, 2016; Gontikaki et al., 2003 | | | |
| *Bittium reticulatum* | | 15 | >35(38.4) | | ***** | 31.5 | Nabozhenko, 2013; Encyclopedia of Life, 2016; Altobelli et al., 2008 | | | |
| *Retusa mammillata* | | 11 | 34 | | 15.0-25.0 | 20 | Zettler and Gosselck 2006; Chaban, 2004; Fleischer and Zettler, 2009 | | | |
| *Chrysallida interstincta* | | 15 | >35 | | ***** | 25 | Funder S. et al., 2002; Rosemberg and Gofas, 2012 | | | |
| ***** No optimal salinity range recovered. | | | | | | | | | |  |

**Extended Data Table S3**—Sample salinity approximation based on weighted averaged salinity optima of the sub-set of the seven key species (Extended Data Table S2) retrieved in each sample. Abbreviation: # number, psu practical salinity unit. Highlighted in red those samples for which species preferred salinity estimate was available for only one or two species representing less 30% of total specimen abundance.

| Core depth (m) | nMDS dataset  sample species richness | # key species available for  salinity computation | % specimens employed for  first approximation sample salinity computation | **Sample** | |
| --- | --- | --- | --- | --- | --- |
|  |  |  |  | **weighted average**  **salinity estimate (psu)** | **nMDS score** |
| 15.60 | 2 | 1 | 1.6 | 21.5 | -0.75963 |
| 12.25 | 7 | 4 | 64.7 | 20.4 | 0.105331 |
| 12.05 | 7 | 5 | 82.4 | 20.9 | 0.238195 |
| 11.95 | 8 | 5 | 61.4 | 24.6 | 0.333406 |
| 11.05 | 6 | 5 | 94.1 | 17.7 | 0.076108 |
| 10.85 | 5 | 4 | 77.8 | 19.7 | -0.24324 |
| 10.25 | 12 | 6 | 72.1 | 23.3 | 0.666585 |
| 10.16 | 9 | 6 | 82.0 | 22.4 | 0.427953 |
| 10.05 | 7 | 5 | 87.0 | 23.0 | 0.07666 |
| 9.90 | 7 | 5 | 78.1 | 22.3 | 0.270657 |
| 9.55 | 3 | 2 | 64.7 | 8.2 | -0.81188 |
| 9.27 | 7 | 4 | 72.7 | 22.6 | 0.246792 |
| 9.05 | 7 | 5 | 88.1 | 23.8 | 0.185443 |
| 8.85 | 10 | 6 | 83.7 | 25.0 | 0.401588 |
| 8.65 | 13 | 5 | 69.0 | 17.1 | 0.26466 |
| 8.40 | 8 | 5 | 75.0 | 19.3 | 0.425677 |
| 8.50 | 7 | 5 | 70.4 | 18.7 | 0.103709 |
| 8.05 | 3 | 2 | 26.7 | 19.0 | -0.74554 |
| 0.85 | 3 | 2 | 50.0 | 12.5 | -0.54542 |
| 0.55 | 6 | 3 | 30.0 | 12.8 | -0.71706 |

Extended Data Table S4—Trematode prevalence data for Abra segmentum from core 204-S7 samples. n = number of valves.

| Core depth (m) | n | n_with trematode pits_ | ntoo fragmented to determine infestation status | Prevalence | Prevalence  _Arcsine-transformed_ |
| --- | --- | --- | --- | --- | --- |
| 18.95 | 4 | 3 | 1 | 0.750 | 1.047 |
| 12.25 | 35 | 17 | 12 | 0.486 | 0.771 |
| 12.05 | 98 | 49 | 22 | 0.500 | 0.785 |
| 11.95 | 39 | 25 | 7 | 0.641 | 0.928 |
| 11.35 | 1 | 0 | 0 | 0.000 | 0.000 |
| 11.05 | 30 | 3 | 9 | 0.100 | 0.322 |
| 10.85 | 14 | 1 | 5 | 0.071 | 0.271 |
| 10.35 | 212 | 111 | 53 | 0.524 | 0.809 |
| 10.16 | 73 | 29 | 24 | 0.397 | 0.682 |
| 9.9 | 51 | 20 | 15 | 0.392 | 0.677 |
| 9.29 | 42 | 29 | 3 | 0.690 | 0.981 |
| 8.85 | 40 | 20 | 7 | 0.500 | 0.785 |
| 8.65 | 27 | 5 | 0 | 0.185 | 0.445 |
| 8.5 | 111 | 21 | 32 | 0.189 | 0.450 |
| 8.4 | 32 | 4 | 6 | 0.125 | 0.361 |
| 0.96 | 3 | 2 | 0 | 0.667 | 0.955 |
| 0.85 | 20 | 8 | 5 | 0.400 | 0.685 |
| 0.55 | 5 | 1 | 1 | 0.200 | 0.464 |

**Extended Data Table S5**—Results of trematode prevalence 10,000 randomization iterations of individual valves. n_valves_ = number of valves examinable in a sample; Prevalence = number of valves with trematode pits divided by n_valves_.

| **Sample**  **core depth (m)** | **n_valves_** | **Prevalence** | **Results of 10,000 randomization iterations of individual valves for trematode prevalence** | | | | |
| --- | --- | --- | --- | --- | --- | --- | --- |
|  |  |  | **Mean** | **0.5%** | **2.5%** | **97.5%** | **99.5%** |
| 0.55 | 5 | 0.200 | 0.413 | 0.000 | 0.000 | 0.800 | 1.000 |
| 0.85 | 20 | 0.400 | 0.415 | 0.150 | 0.200 | 0.650 | 0.700 |
| 8.40 | 32 | 0.125 | 0.415 | 0.219 | 0.250 | 0.594 | 0.656 |
| 8.50 | 111 | 0.189 | 0.416 | 0.297 | 0.324 | 0.505 | 0.541 |
| 8.65 | 27 | 0.185 | 0.416 | 0.185 | 0.222 | 0.593 | 0.667 |
| 8.85 | 40 | 0.500 | 0.416 | 0.225 | 0.275 | 0.575 | 0.625 |
| 9.29 | 42 | 0.690 | 0.417 | 0.238 | 0.262 | 0.571 | 0.619 |
| 9.90 | 51 | 0.392 | 0.415 | 0.235 | 0.275 | 0.549 | 0.588 |
| 10.16 | 73 | 0.397 | 0.414 | 0.274 | 0.301 | 0.534 | 0.575 |
| 10.35 | 212 | 0.524 | 0.415 | 0.330 | 0.349 | 0.481 | 0.505 |
| 10.85 | 14 | 0.071 | 0.414 | 0.071 | 0.143 | 0.643 | 0.786 |
| 11.05 | 30 | 0.100 | 0.415 | 0.200 | 0.233 | 0.600 | 0.633 |
| 11.95 | 39 | 0.641 | 0.416 | 0.231 | 0.256 | 0.564 | 0.615 |
| 12.05 | 98 | 0.500 | 0.416 | 0.286 | 0.316 | 0.510 | 0.541 |
| 12.25 | 35 | 0.486 | 0.415 | 0.200 | 0.257 | 0.571 | 0.629 |
| 18.95 | 4 | 0.750 | 0.407 | 0.000 | 0.000 | 1.000 | 1.000 |


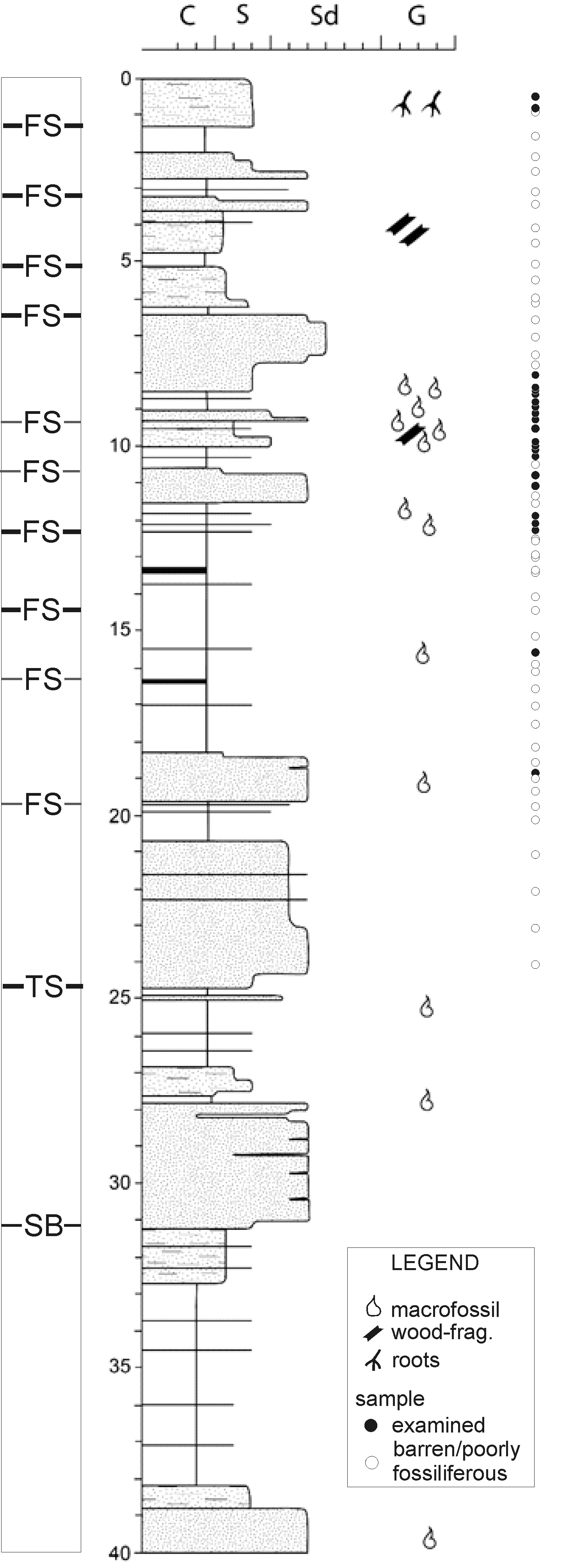


Extended Data Figure 1—204-S7 stratigraphic log showing position of samples, sequence boundary and flooding surfaces (millennial—thick line, and sub-millennial—thin line, time-scales). FS flooding surface; TS transgressive surface, SB sequence boundary.


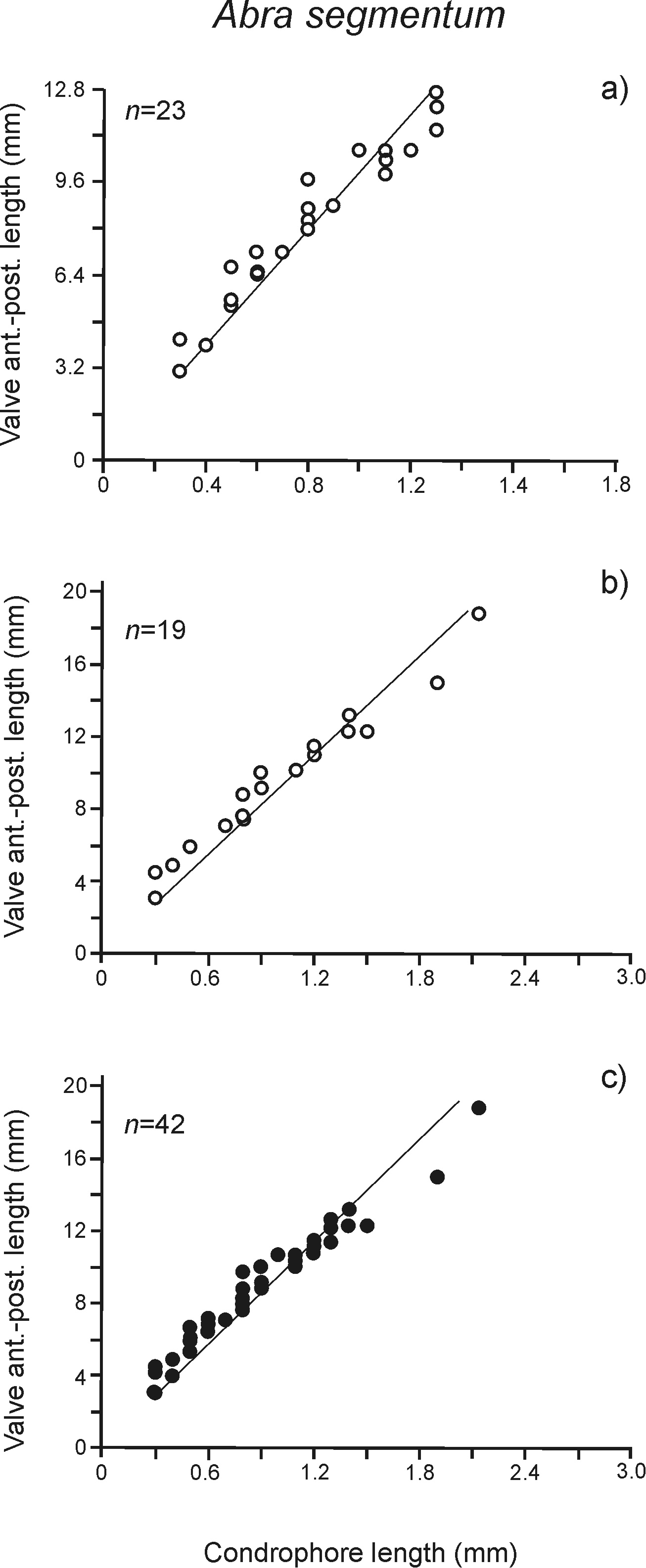


Extended Data Figure 2—Abra segmentum valves were classified as either left or right, and anterior-posterior length (APL) was measured for all complete specimens. In the case of broken valves, we employed the chondrophore length (external portion; Fig. 1C) as a proxy for APL by means of reduced major axis regression (RMA). RMA was performed with intercept centered to 0 value and on a set of (a) 23 right, and (b) 19 left valves, and (c) a set of all valves measured. The RMA model of all valves was utilized for estimating APL of fragmented valves whose designation as a right or left valve was not possible. Estimation of anterior-posterior length was not employed for fragmented valves lacking the umbonal region. A) RMA regression for right valves. Regression coefficient and tests: Pearson r = 0.97; r^2^ = 0.94; p(uncorr.) <<0.05; Slope 9.9566 (95% bootstrapped C.I. 9.46-10.34 based on 1999 iterations), Standard error 0.212. B) RMA regression for right valves. Regression coefficients and tests: Pearson r = 0.99; r^2^ = 0.97; p(uncorr.) <<0.05; Slope 9.1483 (95% bootstrapped C.I. 8.54-9.57 based on 1999 iterations), Standard error 0.232. C) RMA regression for all valves. Regression coefficient and tests: Pearson r = 0.98; r^2^ = 0.96; p(uncorr.) <<0.05; Slope 9.5008 (95% bootstrapped C.I. 9.06-9.86 based on 1999 iterations), Standard error 0.16659. Ant.=Anterior; Post.=posterior


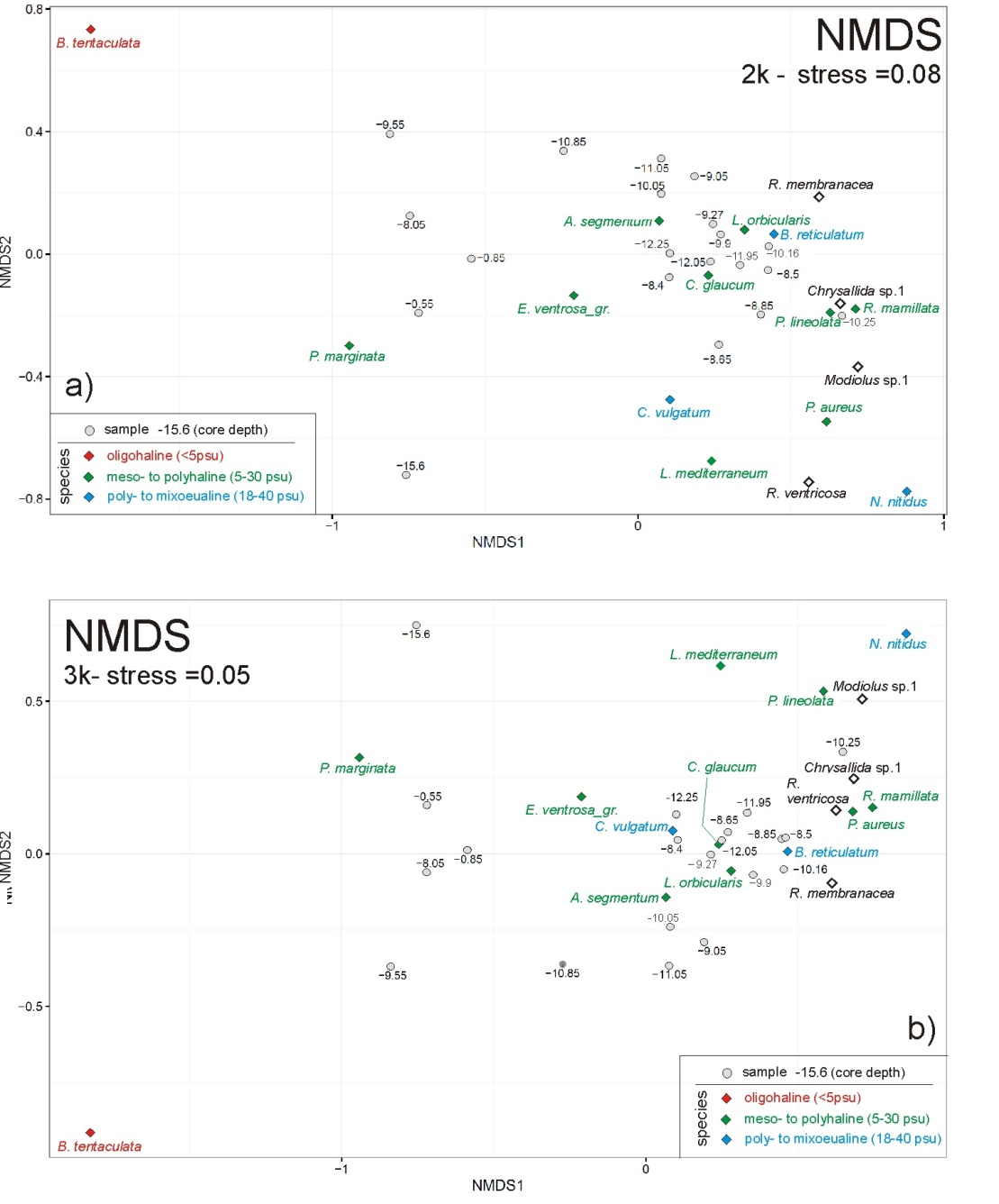


Extended Data Figure 3—Non-metric Multidimensional Scaling (nMDS) ordination was used to investigate the primary environmental gradient along which the samples could be ordinated. Only small samples (n<15 specimens), singletons (1 occurrence), and congeneric specimens without species designations removed. Bray-Curtis similarity was used to develop nMDS ordinations; both (a) 2 dimension and (b) 3 dimension outputs are reported. In both outputs, the indirect ordination analyses of the core data revealed a pronounced gradient, with species ordinated along the first nMDS axis, according to their salinity tolerance.


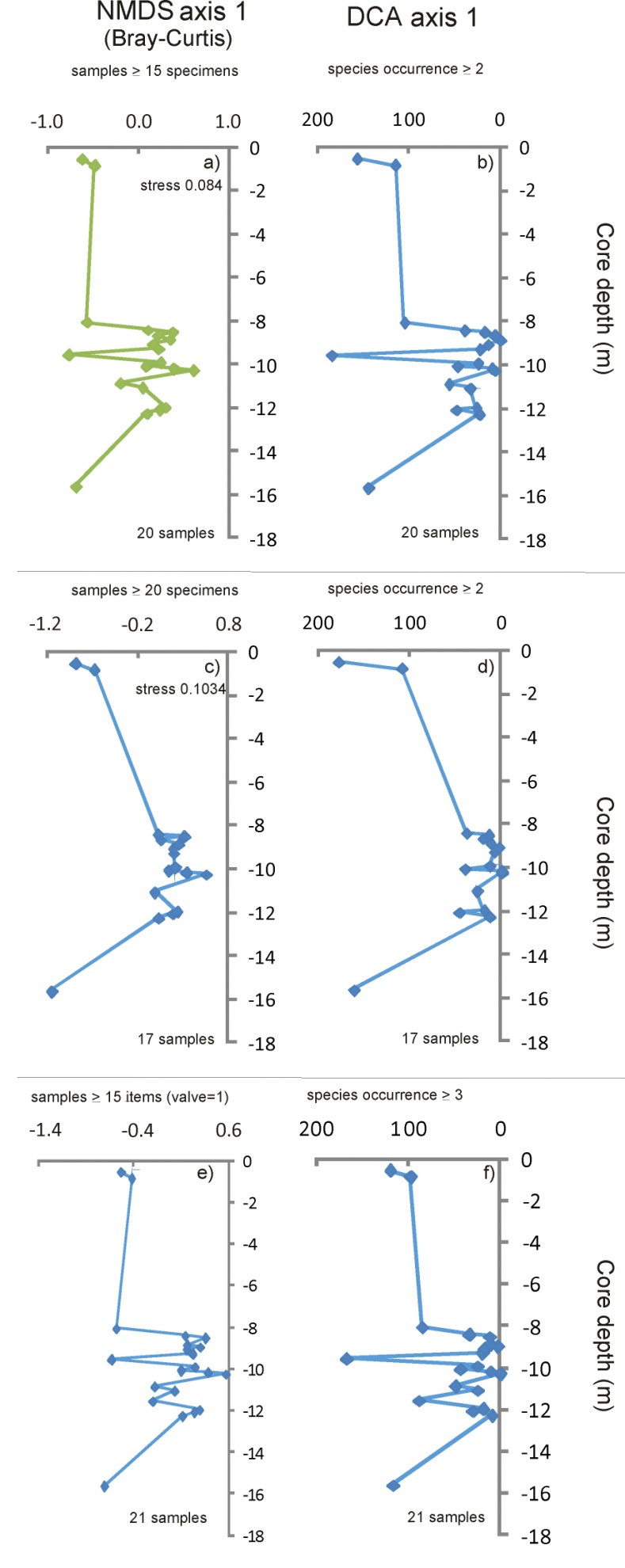


Extended Data Figure 4—non-metric Multidimensional Scaling (nMDS) and Detrended Correspondence Analysis ordinations varying rare taxa or sample thresholds returned comparable patterns. A-b) Matrix log-transformed, minimum sample size ≥15 specimens (valves counted as 0.5), only species occurring in one sample and samples with one species were excluded. C-d) As above, but minimum sample size ≥20 specimens (valves counted as 0.5). E-f) Matrix log-transformed, minimum sample size ≥15 specimens (valves counted as 1), only species occurring in less than three samples and samples with one species were excluded. The nMDS profile in green is reported in Figure 2.


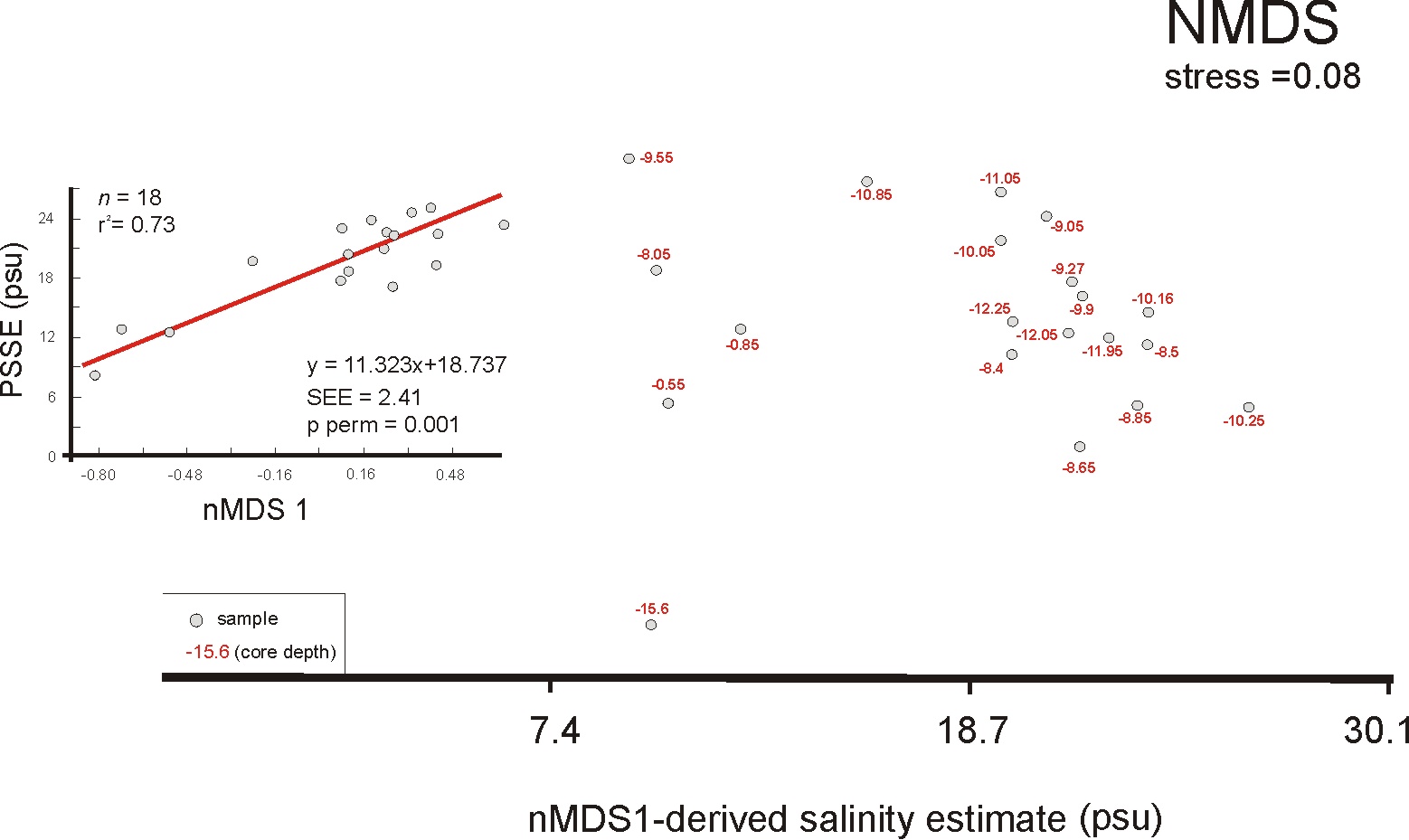


**Extended Data Figure 5**—The species preferred salinity was independently estimated using the modern ecological data for mollusk (Extended Data Tables S2-3), and then correlated with the sample nMDS score along axis 1. Indeed, a recent study on comparable successions and based on averaging and Detrended Correspondence approaches, demonstrated that a posteriori ordination scores are a robust predictor of salinity for both samples and species across Holocene paralic succession of the Arno Plain (see Amorosi et al. 2014). The reduced major axis (RMA) linear regression (red line) is shown. The thick horizontal bottom line represents nMDS sample scores (Fig. Extended Data S3A) calibrated by means of RMA linear regression function. Abbreviations: nMDS1 = non Metric Multidimensional Scaling axis one; n = sample size; pnon param = permutation test on correlation; psu = practical salinity unit; r2 = coefficient of determination; SEE = standard error of the estimate; PSSE = provisory sample salinity estimate (see Extended Data Table S3).


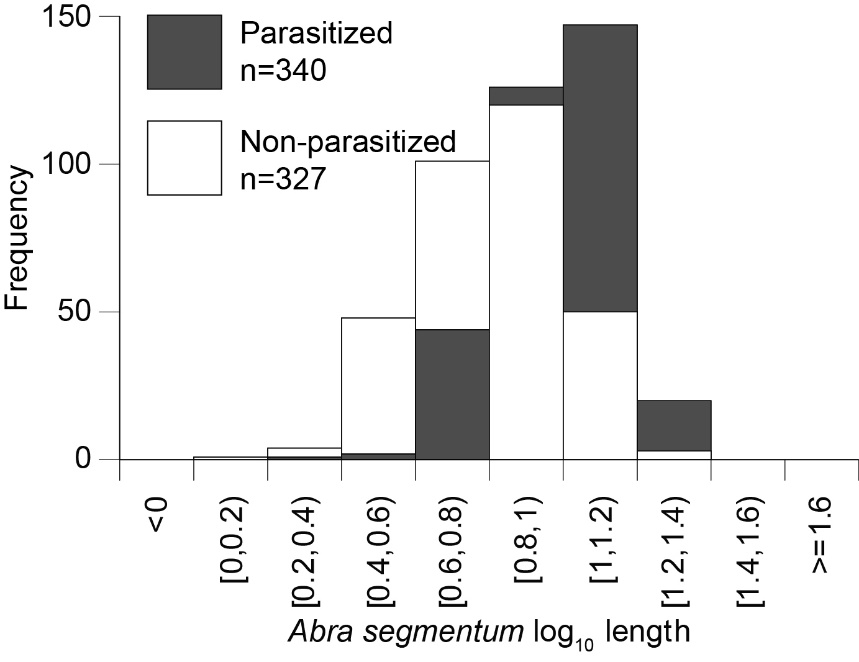


**Extended Data Figure 6**—Size frequency distribution of *Abra segmentum* recovered in core 204-S7 classified by presence/absence of trematode-induced pits.

Extended Data References

Altobelli, A., Hubina, T., Sponza, S., & Sisto, A. Effect of abiotic and biotic factors on the abundance of waterbirds in Grado-Marano Lagoon (Italy). In SPIE Remote Sensing, pp. 710404-710404 (2008, October). International Society for Optics and Photonics.

Amorosi, A., Centineo, M. C., Colalongo, M. L., & Fiorini, F. Millennial-scale depositional cycles from the Holocene of the Po Plain, Italy. Marine Geology, 222, 7-18 (2005).

Amorosi, A., Bruno, L., Rossi, V., Severi, P., & Hajdas, I. Paleosol architecture of a late Quaternary basin–margin sequence and its implications for high-resolution, non-marine sequence stratigraphy. Global and Planetary Change, 112, 12-25 (2014).

Amorosi A. et al. Global sea-level control on local parasequence architecture from the Holocene record of the Po Plain, Italy. Marine and Petroleum Geology (2017).

Anderson, M. J. et al., S. P. Navigating the multiple meanings of β diversity: a roadmap for the practicing ecologist. Ecology letters, 14(1), 19-28 (2011).

Anistratenko, V. V., Khaliman, I. A., & Anistratenko, O. Y. Molluscs the Sea of Azov. Kiev: Naukova Dumka (2011).

Bambach, R. K., & Kowalewski, M. How to count fossils. In Geological Society of America Abstracts with Programs, 32(7), 332 (2000).

Berezina, N.A. Tolerance of Freshwater Invertebrates to Changes in Water Salinity. Russian Journal of Ecology 34, 261-266 (2003).

Boyden, C. R., & Russell, P. J. C. The distribution and habitat range of the brackish water cockle (Cardium (Cerastoderma) glaucum) in the British Isles. The Journal of Animal Ecology, 719-734 (1972).

Bruno, L., Amorosi, A., Severi, P., & Costagli, B. Late Quaternary aggradation rates and stratigraphic architecture of the southern Po Plain, Italy. Basin Research (2016).

Caspian Sea Biodiversity (CSB) Database. Accessed thorough: http://www.caspianenvironment.org/biodb/eng/main.htm on 07/05/2016.

Chaban, E.M. Some materials for revision of opistobranchs of the family Retusidae (Mollusca:Cephalaspidea). Annual Reports of the Zoological Institute Russian Accademy of Sciences (2004).

Cilenti, L. et al. Population structure and spatial distribution of Loripes lacteus (Linnaeus, 1758) in Varano lagoon, SE Italy. Transitional Waters Bulletin, 2(4), 63-70 (2010).

Der, G., & Everitt, B. S. Essential Statistics Using SAS University Edition. Sas Institute (2015).

Fleischer, D., Zettler, M.L. An adjustment of benthic ecological quality assessment to effects of salinity. Marine Pollution Bulletin 58, 351-357 (2009).

Funder, S., Demidov, I., Yelovicheva, Y. Hydrography and mollusc faunas of the Baltic and the White Sea-North Sea seaway in the Eemian. Palaeogeography, Palaeoclimatology, Palaeoecology, 184, 275-304 (2002).

Gontikaki, E., Antoniadou, C., Chintiroglou, C.C. Population structure of Cerastoderma glaucum and Abra ovata in Vouliagmeni Lagoon (Attiki). Journal of Marine Biological Associations of the United Kingdom 83, 1095-1097 (2003).

Hammer, Ø., Harper, D.A.T., and P. D. Ryan. PAST: Paleontological Statistics Software Package for Education and Data Analysis. Palaeontologia Electronica 4(1): 9pp (2001).

Nabozhenko. Reconstruction and dinamics of bivalvia taxocenosis (Mollusca: Bivalvia) of the Sea of Azov in the late Holocene in connection with change of salinity. Proceedings of the Zoological Institute of the Russian Academy of Sciences Application No. 3, 2013, c. 182-191 (2013).

Neal, J., & Abreu, V. Sequence stratigraphy hierarchy and the accommodation succession method. Geology, 37(9), 779-782 (2009).

Rosenberg, G., Gofas, S. Chrysallida interstincta (J. Adams, 1797) (2012). Accessed through: World Register of Marine Species at http://www.marinespecies.org/aphia.php?p=taxdetails&id=140918 on 2012-05-11.

R Core Team. R: A language and environment for statistical computing. R Foundation for Statistical Computing, Vienna, Austria (2016). URL https://www.R-project.org/.

Scarponi, D., Angeletti, L. Integration of paleontological patterns in the sequence stratigraphy paradigm: A case study from Holocene deposits of the Po Plain (Italy). GeoActa, 7, 1-13 (2008).

Scarponi, D., Kaufman, D., Amorosi, A. and Kowalewski, M. Sequence stratigraphy and the resolution of the fossil record. Geology, 41/2, 239–242 (2013).

Scarponi, D. et al. Systematic vertical and lateral changes in quality and time resolution of the macrofossil record: insights from Holocene transgressive deposits, Po coastal plain, Italy. Marine and Petroleum Geology (submitted).

Zettler, M.L., Gosselck, F. Benthic assessment of marine areas of particular ecological importance within the German Baltic Sea EEZ. In: Progress in Marine Conservation in Europe - Natura 2000 sites in German offshore waters. H. von Nordheim, D. Boedeker, J.C. Krause (eds.) Springer Berlin (Chapter 8) (2006).
